# Preoperative glycaemic control, number of pain locations, structural knee damage, self‐reported central sensitisation, satisfaction and personal control are predictive of 1‐year postoperative pain, and change in pain from pre‐ to 1‐year posttotal knee arthroplasty

**DOI:** 10.1002/ksa.12265

**Published:** 2024-05-15

**Authors:** Sophie Vervullens, Lotte Meert, Rob J. E. M. Smeets, Jonas Verbrugghe, Isabel Baert, Frank Th. G. Rahusen, Christiaan H. W. Heusdens, Peter Verdonk, Mira Meeus

**Affiliations:** ^1^ Research Group MOVANT, Department of Rehabilitation Sciences and Physiotherapy (REVAKI) University of Antwerp Wilrijk Belgium; ^2^ Research School CAPHRI, Department of Rehabilitation Medicine Maastricht University Maastricht The Netherlands; ^3^ Pain in Motion International Research Group (PiM), www.paininmotion.be Antwerp Belgium; ^4^ CIR Clinics in Revalidatie Eindhoven The Netherlands; ^5^ REVAL‐Rehabilitation Research Center, Faculty of Rehabilitation Sciences Hasselt University Hasselt Belgium; ^6^ Department of Orthopaedics St Jans Gasthuis Weert The Netherlands; ^7^ Department of Orthopedics and Traumatology University Hospital of Antwerp Antwerp Belgium; ^8^ Faculty of Medicine and Health Sciences University of Antwerp Antwerp Belgium; ^9^ ORTHOCA Antwerp Belgium; ^10^ ASTARC Department Antwerp University Antwerp Belgium

**Keywords:** chronic postoperative pain, knee osteoarthritis, prognostic factors, total knee arthroplasty

## Abstract

**Purpose:**

The aim of this study was to identify preoperative predictors for 1‐year posttotal knee arthroplasty (TKA) pain and pre‐ to post‐TKA pain difference in knee osteoarthritis (KOA) patients.

**Methods:**

From March 2018 to July 2023, this prospective longitudinal cohort study enrolled KOA patients awaiting TKA from four hospitals in Belgium and the Netherlands. Different biopsychosocial predictors were assessed preoperatively by questionnaires and physical examinations (input variables). The Knee injury and Osteoarthritis Outcome Score (KOOS) subscale pain was used to measure pain intensity. The absolute KOOS subscale pain score 1‐year post‐TKA and the difference score (ΔKOOS = 1‐year postoperative − preoperative) were used as primary outcome measures (output variables). Two multivariable linear regression analyses were performed.

**Results:**

Two hundred and twenty‐three participants were included after multiple imputation. Worse absolute KOOS subscale pain scores 1‐year post‐TKA and negative or closer to zero ΔKOOS subscale pain scores were predicted by self‐reported central sensitisation, lower KOA grade and preoperative satisfaction, and higher glycated haemoglobin, number of pain locations and personal control (adjusted *R* ^2^ = 0.25). Additional predictors of negative or closer to zero ΔKOOS subscale pain scores were being self‐employed, higher preoperative pain and function (adjusted *R* ^2^ = 0.37).

**Conclusion:**

This study reports different biopsychosocial predictors for both outcomes that have filtered out other potential predictors and provide value for future studies on developing risk assessment tools for the prediction of chronic TKA pain.

**Protocol Registration:**

The protocol is registered at clinicaltrials.gov (NCT05380648) on 13 May 2022.

**Level of Evidence:**

Level II.

## Graphical abstract

Self‐reported symptoms of central sensitisation, higher glycated haemoglobin, higher preoperative satisfaction, less structural knee damage, higher number of pain locations and better personal control were consistent preoperative predictors of both more pain 1 year after total knee arthroplasty and a pain deterioration or less pain improvement 1 year after total knee arthroplasty.

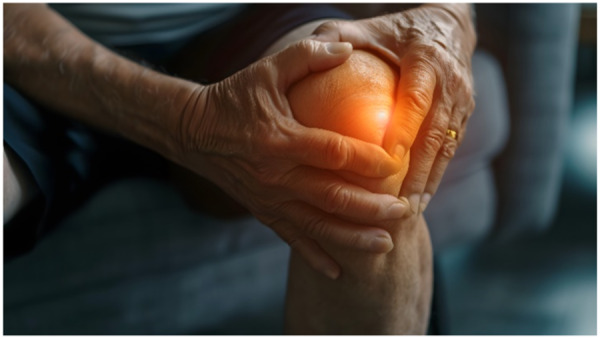

**Abbreviations**
ANOVA: analysis of variance
BMI: body mass index
CI: confidence interval
CPM: conditioned pain modulation
CRP: C‐reactive protein
CSI: Central Sensitisation Inventory
ECRL: musculus extensor carpi radialis longus
HADS: Hospitality Anxiety And Depression Scale
HbA1c: glycated haemoglobin
IPQR: Illness Perceptions Questionnaire Revised
KOA: knee osteoarthritis
KOOS: Knee injury and Osteoarthritis Outcome Score
KSSS: Knee Society Scoring System
M2SENS: Sensoric Functioning Lab
PCS: Pain Catastrophising Scale
PPT: pressure pain threshold
STROBE: Strengthening The Reporting of Observational studies in Epidemiology
TKA: total knee arthroplasty
ΔKOOS: difference in KOOS score preoperative versus 1‐year postoperative

## INTRODUCTION

Despite the generally high success rate of total knee arthroplasty (TKA), approximately 20% of patients experience chronic postoperative pain [4, 44, 61]. Understanding and identifying factors associated with chronic TKA pain is crucial to identify causal predictors, which could optimise interventions and facilitate stratified care [41, 61].

A recent umbrella review of 18 systematic reviews synthesised all‐potential preoperative predictive factors for chronic postoperative pain after TKA or total hip arthroplasty [16]. The identified factors encompassed the entire biopsychosocial model (Table 1). However, as this was an umbrella review, distinguishing findings of multivariable and univariate analyses was not convenient [16]. Univariate analyses reveal potential predictive factors (i.e. factors associated with a certain outcome) but should not be confused with definitive predictors or causal factors. To achieve the latter, consistent findings of high‐quality multivariable regression models are necessary. This enables distinguishing the real predictive factors that may ‘filter out’ other factors [41, 52].

**Table 1:** Prognostic factors of postsurgical pain with confidence in conclusion level according to the umbrella review of Fernández‐de‐Las‐Peñas et al. [16].

Variable	High/moderate confidence in conclusion for association with worse postoperative pain	High/moderate confidence in conclusion for no association with worse postoperative pain	Low/very low confidence in conclusion for association with worse postoperative pain	Low/very low confidence in conclusion for no association with worse postoperative pain	Conflicting or not possible to draw a conclusion
Demographic factors	African‐American ethnicity				Age, gender
Structural variables			Less radiographic damage, presence of preoperative flexion contracture		
Metabolic variables			Presence of diabetes mellitus		BMI
Functional variables	Poor function			Lower ROM	
Pain‐related variables	Pain at other sites, higher pain severity, the presence of neuropathic pain, disturbed somatosensory functioning, opioid use				
Psychological variables	Higher level of pain catastrophising, anxiety, depression, fear of movement and worse mental health and coping				Having purpose in life, psychological distress, patient expectations, quality of life, self‐efficacy
Social variables	Lower social support				Educational level, socioeconomic status, personality (optimistic or pessimistic)
Comorbidities		Heart and lung disease, stroke, nervous system disorders such as Alzheimer's disease, Parkinson's disease, dementia and poor blood circulation	Contralateral hip osteoarthritis		Kidney disease, low back pain
Other variables					Length of the waiting list

Abbreviations: BMI, body mass index; ROM, range of motion.

Fortunately, a recent systematic review and meta‐analysis of factors associated with post‐TKA pain presented a distinction between the results of univariate and multivariable analyses [18]. Only higher state anxiety and depression had a consistent bidirectional univariate association with persistent post‐TKA pain, whereas higher preoperative pain severity was the only independent predictive factor based on all multivariable analyses. The authors emphasise that current findings are still of low evidence and based on limited data, warranting more research. Moreover, multicentre prospective studies that comprehensively combine a broad range of biopsychosocial possible predictors into one multivariable analysis are scarce [18], with the study of Edwards et al. [14] prediction being the only one up till now.

Despite the significant contribution of Edwards et al. [14], only potential predictors of 6‐month post‐TKA pain were studied, while a recovery period of 1 year is regarded essential for complete recuperation after TKA [47]. This makes more elaborative research in this domain necessary to offer valuable insights for future studies in identifying causal predictors for chronic post‐TKA pain, which in turn could improve the quality of care for TKA by developing consistent clinical prediction models. It is for instance postulated that prehabilitation may improve postsurgical outcomes when targeting modifiable causal predictive factors with post‐TKA pain [41, 56].

Thus, the aim of this prospective, multicentre longitudinal study was to determine preoperative predictors for 1‐year post‐TKA pain and difference in pain from pre‐ to post‐TKA in knee osteoarthritis (KOA) patients. These predictors, encompassing the entire biopsychosocial model, were analysed using two multivariable linear regression models.

## METHODS

Strengthening The Reporting of Observational studies in Epidemiology (STROBE) guidelines for cohort studies were used to conduct this longitudinal prospective cohort study [58]. The protocol is registered at clinicaltrials.gov (NCT05380648).

### Setting and participants

This multicentre prospective cohort study was conducted from March 2018 to July 2023 (recruitment period between March 2018 and July 2022 followed by 1‐year data collection). Patients with KOA awaiting TKA were recruited at the University Hospital of Antwerp and AZ Monica in Belgium, and the Academic Hospital of Maastricht and St. Jans Gasthuis Weert in the Netherlands. The ethical committees of both countries approved the study (BE300201319366 and NL6465408618, respectively).

Participants were eligible if diagnosed with KOA, awaiting their first TKA and aged 40 years or older. Exclusion criteria included experiencing neurological or systemic diseases that could potentially impact pain perception or the inability to speak or understand Dutch. After providing informed consent, participants completed demographic, psychological, functional, and symptom‐related questionnaires, as described below, either on paper or online via Qualtrics (www.qualtrics.com). Two executive researchers, S. V. or L. M., conducted the physical measurements at the Sensoric Functioning Lab (M2SENS) at the University of Antwerp's campus ‘Drie Eiken’ for Belgian participants and at the orthopaedic department of the Academic Hospital Maastricht and St. Jans Gasthuis Weert for Dutch participants. Both researchers fulfilled practical skills training and used standardised measurement forms. Data were collected 4 weeks before TKA surgery (baseline) and 1‐year post‐TKA. All individuals were instructed to stop early‐stage pain medications, coffee and alcohol 24 h before physical evaluations.

### Outcome variable

The ‘pain’ subscale of the Knee injury and Osteoarthritis Outcome Score (KOOS) was utilised as the primary outcome measure for pain intensity 1 year after surgery. Scores were converted to percentages and ranged from 0 (*extreme pain*) to 100 (*no pain*) [42]. The absolute KOOS subscale pain score 1‐year post‐TKA and the difference in KOOS subscale pain score preoperative versus 1‐year postoperative (ΔKOOS = postoperative − preoperative) were used as outcome measures. A negative score or a score closer to zero of ΔKOOS subscale pain was interpreted as a more insufficient outcome.

### Possible predictors

All potential predictors were prospectively collected 4 weeks prior to the TKA surgery, except for C‐reactive protein (CRP), which was retrospectively extracted from the patients' medical records. A list of these possible predictors, along with their respective measurement methods and clinimetric properties, can be found in Table 2.

**Table 2:** Overview of possible predictors (bibliography of references can be found in Supporting Information S2: Resource 2).

Variable	Measurement method	Measurement device Data type Scoring Reference to psychometric properties
Demographic variables		
Age	Date first physical measurement—Birth date	Demographic questionnaire Continuous variable
Sex	Man or woman	Demographic questionnaire Nominal variable
Structural factors		
Grade of KOA	X‐ray images in AP, profile and Rosenberg weight‐bearing position [30] Retrospectively extracted from the participant's record by the general practitioner of the participants or the participants themselves If one of the images was not available, scoring was based on the available image(s). If no x‐ray image was available, MRI in coronal and sagittal positions was extracted and MRI grading was transferred to K&L grading If none of the x‐ray or MRI images could be found, this variable was recorded as a missing value. All images were scored by the same orthopaedic surgeon (C. H. W. H.)	K&L scale [15] or MRI grading system [37] Ordinal variable 5‐point Likert scale: 0 (*no KOA*) to 4 (*worst grade of KOA*) K&L: Good reliability and validity in KOA [45] MRI grading: Good reliability and responsiveness [22]
Metabolic and inflammatory factors		
BMI	Length: Self‐reported Weight: Standing on an electronic scale at the moment of testing	Length: Demographic questionnaire; weight: electronic scale Continuous variable Formula: kg/cm^2^ Valid [21]
HbA1c	Sitting position Taking a blood sample by pricking into a fingertip	A1CNow+ system (PTS Diagnostics) and a fingerprick [39] Continuous variable Percent Accurate measurement to detect diabetes [48]
Fat mass	Supine lying position Skinfold electrodes on hand and foot connected to the device	Bioelectrical Impedance Analysis (Bodystat Quadscan 4000) Continuous variable Percent Accurate measurement to measure body composition [13]
Lean mass		
C‐reactive protein	Blood sample before surgery, retrospectively extracted from participant's record by executive researchers	Blood sample Continuous variable mg/L Reliable method [50]
Functional variables		
Strength musculus quadriceps	Sitting position with hip and knee in 90°, upper leg fully supported by the table, and arm crossed over their chest. Isometric strength measurement was assured by using a traction belt Perform flexion (hamstrings) or extension (quadriceps) of the knee against the device Three times, highest value used for analysis	MicroFET 2 hand‐held dynamometer (ProCare) Continuous variable kgf Reliable and valid [27]
Strength musculus hamstrings		
Proprioception	Sitting position with hip and knee in 90°, upper leg fully supported by the table Repositioning error during a knee joint position sense test (20°, 45° and 70° flexed knee) Twice assessed, mean of six trials used for analysis	Plurimeter (Dr. Rippstein) Continuous variable Degree of knee angle Reliable [5]
Functional symptoms	Questionnaire: Questions related to stiffness, noises and mobility of the knee	KOOS subscale symptoms Continuous variable 5‐point Likert scale: 0 (*no symptoms*) to 4 (*always symptoms*) for questions 1 to 5, 4 (*always*) to 1 (*never*) for questions 6 and 7. Scores were converted to a 0–100 scale, ranging from 0 (*extreme knee problems*) to 100 (*no knee problems*) Valid and reliable [9]
Physical function	Questionnaire: Asking questions related to different activities	KSSS functional score Continuous variable Scored 0 (*impossible to perform any activities*)–120 (*possible to perform any activity*); sum of subscales ‘walking and standing’, ‘standard activities’, ‘advanced activities’ and ‘discretionary activities Valid and reliable [32]
	Sitting position with arms resting next to the body Standing up and again sitting down as much as possible without support in 30 s	30 CST Continuous variable Number of times to stand up Reliable [19]
Pain‐related variables		
Pain intensity	Questionnaire: Questions related to pain intensity and specific movements during previous months	KOOS subscale pain Continuous variable 5‐point Likert scale: 0 (*no pain*) to 4 (*unbearable pain*); scores were converted to a 0–100 scale, ranging from zero (*extreme pain*) to 100 (*no pain*) Valid and reliable [9]
Number of pain locations	To draw their pain on a body chart by crossing all body parts that were painful during the last week	Pain drawings on body chart Continuous variable Number of body parts Valid and reliable [46]
Somatosensory functioning		
Pressure pain thresholds	A probe (1 cm^2^) was placed perpendicular to the test surface and pressure was increased until the subject reported a feeling of discomfort. Measured at the medial and lateral knee‐joint line, and m. Tibialis anterior of the affected knee, the m. ECRL) of the nondominant side and the forehead	Hand‐held pressure algometer (Wagner FDX 25 Force Gage) Continuous variable An average of two measurements, separated by a pause of 30 s, was taken for analysis (Newton) Reliable [63]
Temporal summation and painful after sensations	Thirty pinpricks were given at a pace of 1 pinprick/second. Measured at the medial knee‐joint line and medial wrist of the affected side	Von Frey monofilament (60 g) Continuous variable NRS score 0–10 Reliable [8, 10]
Heat and cold allodynia	A roll movement was performed for 10 s at the medial and lateral knee‐joint line of the affected knee, and the m. Extensor carpi radialis longus of the nondominant side	Thermal rollers (Rolltemp II Somedic Senselab) having a temperature of 25°C (cold stimulus) and 40°C (hot stimulus) Continuous NRS score 0–10 Reliability unknown
Conditioned pain modulation	First, a temperature corresponding to a pain intensity NRS score of 4/10 (up to a maximum of 46°C) was identified at the wrist of the affected side. This identified temperature (or 46°C when the 4/10 on a NRS was not reached) was used as test stimulus. The participant had to score the test stimulus on an NRS four times. After a pause of 120 s, a conditioning stimulus (with a temperature of 0.5°C more than the test stimulus) was added at the wrist of the nonaffected side for 65 and 20 s after its initiation, the test stimulus was repeated. Again, the participants had to score their pain for four times, but only on the test site. If the NRS at 46°C and the mean of the NRS of test stimulus was equal to 0, the participant was excluded for analysis	Q‐sense CPM (Medoc) Continuous variable NRS: 0 (*no pain*) to 10 (*unbearable pain*); percentage change ((absolute score/NRS score during test stimulus) × 100) scores were used for analysis Reliability to better confirmed [10]
Sensitisation‐associated symptoms	Questionnaire: Assesses self‐reported central sensitisation signs in 25 questions	CSI Continuous variable Five‐point Likert scale with 0 meaning ‘*never*’ and 4 meaning ‘*always*’; score from 0 to 100 Reliable [25]
Psychological variables		
Pain catastrophising	Questionnaire: Questions related to pain catastrophising Three subdomains: Magnification, rumination and helplessness	PCS Continuous variable 5‐point Likert scale: 0 (*not at all*) to 4 (*all the time*); total score was used for the analysis Valid and reliable [33, 34]
Depression	Questionnaire: Questions related to depression and anxiety Two subscales: Depression and anxiety	HADS Continuous variable 4‐point Likert scale: 0–3 (variable meaning per item); scores of two subscales were used for analysis Valid and reliable [51]
Anxiety		
Expectations	Questionnaire: Questions related to surgery result expectation Subscale ‘expectations’ was used for analysis	KSSS Continuous variable 6‐point Likert scale: 0 (*no expectation*) to 5 (high positive expectations) Valid and reliable [32]
Satisfaction	Questionnaire: questions related to satisfaction about knee complaint Subscale ‘satisfaction’ was used for analysis	KSSS Continuous variable Five items scored from 0 (*no expectation*) to 8 (*high positive expectations*) Valid and reliable [32]
Consequences	Questionnaire: Questions related to consequences of KOA complaint	IPQR Continuous variable Six items scored from 1 (*strongly disagree*) to 5 (*strongly agree*); subscale identity scored differently: 9 symptoms related to illness scored 0 (*no*) or 1 (*yes*) Reliable, except for subscale coherence [26]
Timeline	Questionnaire: Questions related to timeline of KOA complaint	
Timeline cyclical		
Personal control	Questionnaire: Questions related to personal control over the KOA disease	
Treatment control	Questionnaire: Questions related to treatment control over the KOA treatment	
Emotional representation	Questionnaire: Questions related to emotional representation	
Illness coherence	Questionnaire: Questions related to illness coherence	
Identity	Questionnaire: Questions related to experienced symptom related (or not) to the disease	
Social variables		
Work	Questionnaire: Questions related to work level including pension, self‐employed, white‐collar worker, labourer, unemployed or other	Demographic questionnaire Nominal variable Scored from 1 to 6
Education	Questionnaire: Questions related to educational level going from no degree, primary school degree, technical secondary school degree, higher secondary school degree, high school degree, university degree to other	Demographic questionnaire Nominal variable Scored from 1 to 7
Marital status	Questionnaire: Questions related to marital status including married, divorced, single, widow(er) or other	Demographic questionnaire Nominal variable Scored from 1 to 5

Abbreviations: 30CST, 30 s timed chair stand test; AP, anterior–posterior; BMI, body mass index; CPM, conditioned pain modulation; CSI, Central Sensitisation Inventory; ECRL, extensor capri radialis longus; HADS, Hospital Anxiety and Depression Scale; HbA1c, glycated haemoglobin; IPQR, Illness Perception Questionnaire Revised; kgf, kilogram force; K&L scale, Kellgren and Lawrence scale; KOA, knee osteoarthritis; KSSS, Knee Society Scoring System; MRI, magnetic resonance images; KOOS, Knee Osteoarthritis Outcome and Index Score; N/A, not applicable; NRS, numeric rating scale; PCS, pain catastrophising scale; SPS, somatosensory processing signs.

### Statistical analysis

The R software (version 4.2.3) (multiple imputation) and the IBM Statistical Package for Social Sciences Version 29 (SPSS; IBM Corporation) (all other statistical analyses) were used.

First, univariate outliers were checked using boxplots and only deleted if due to data input mistakes. Thereafter, missing data were checked, and multiple imputation (*n* = 10 imputed data sets) using predictive mean matching with the ‘mice’ package in R was performed for data with <40% missing [23]. Data were presented as mean and standard deviation (continuous demographic data) and number and frequency (categorical demographic data). Rubin's rules were applied to pool all data.

Next, the assumption of multicollinearity was checked with univariate association analyses using Pearson's correlation (normal and linear distributed data) and Wilcoxon's rank‐sum tests (nonnormal and nonlinear distributed data) between the possible predictors. When variables were highly correlated (correlation coefficient *r* ≥ 0.70 or ≤−0.70), only one was chosen to include for further analyses based on expertise. In addition, the variance inflation factor was checked, and if >4, the variable was deleted from the analysis [20].

Last, a multivariable regression analysis of variance analysis (ANOVA) was performed for each outcome variable. Univariate associations between the two outcomes and the possible predictors were checked using Pearson's correlation (normal and linear distributed data) and Wilcoxon's rank‐sum tests (nonnormal and nonlinear distributed data), and variables with a *p* value <0.2 were included at the start of the multiple regression ANOVA to ensure that the rule of thumb of a maximum of 1 predictor per 10 subjects was met [20]. If nonlinearity with one of the outcome variables was present, this variable was transformed to a categorical variable, a logarithmic value or a Box–Cox transformation to meet this assumption. Also, normality and homogeneity of variance of the residuals were checked using histograms and scatterplots. Backward selection was performed using the median *p* values of the 10 imputed data sets [7]. If this *p* value was <0.05, the variable was kept in the model. All results were pooled using Rubin's rules, and median *p* values for the 10 imputed data sets were reported [7].

### Sample size

All eligible candidates during the period of March 2018 to July 2022 were included, based on a priori sample size calculation of another study of the project [57]. The rule of a minimum of 10 subjects per predictor was used to define the number of predictors in the multivariable linear regression models [20].

## RESULTS

### Participants

A total of 223 participants were analysed after multiple imputation, of which 18 participants were tested for more than 4 weeks preoperatively due to coronavirus disease 2019 surgery postponement. However, these 18 reported no difference compared to other participants in ΔKOOS subscale pain or the absolute score at 1‐year post‐TKA (*p* > 0.05). Fifty‐three (23.7%) participants underwent surgery in the Netherlands (2 [1%] in the University Hospital of Maastricht, and 51 [23%] in SJG Weert), and 170 (76.3%) in Belgium (41 [18%] in the University Hospital of Antwerp and 129 [58%] in AZ Monica). All demographic, baseline values and outcome scores are presented in Table 3.

**Table 3:** Demographics, baseline and outcome scores of participants.

Continuous variables			Categorical variables		
Variable	Mean (SD)	*N* missing (%)	Variable	*N* (%)	*N* missing (%)
*Demographic variables*			*Demographic variables*		
Age (years)	65.52 (7.66)	0 (0)	Sex (*n*, % F)	111 (49.8)	0 (0)
*Metabolic and inflammatory variables*			*Structural variables*		
BMI (kg/cm^2^)	29.99 (5.25)	3 (1.3)	K&L scale		9 (4)
HbA1c (%)	5.60 (0.60)	21 (9.4)	1	4 (1.8)	
Fat (%)	35.15 (8.88)	91 (40.8)	2	44 (19.7)	
Lean (%)	64.85 (8.88)	91 (40.8)	3	77 (34.5)	
C‐reactive protein (mmol/dL)	3.51 (4.83)	146 (65.5)	4	89 (39.9)	
			*Social variables*		
Functional variables			Marital status		10 (4.5)
Strength m. Quadriceps (kgf)	27.37 (13.04)	3 (1.3)	Married	125 (68.2)	
Strength m. Hamstrings (kgf)	11.73 (5.94)	3 (1.3)	Divorced	20 (9.0)	
Proprioception (°)	4.44 (2.04)	6 (2.7)	Single	8 (3.6)	
KOOS symptoms (0–100)	48.89 (18.06)	12 (5.4)	Widow(er)	19 (8.5)	
30CST (*n*)	10.66 (3.97)	6 (2.7)	Other	14 (6.3)	
KSSS functional score (0–100)	43.07 (15.17)	14 (6.3)			
			Work		10 (4.5)
Pain‐related variables			Pension	115 (51.6)	
KOOS subscale pain BL (0–100)	44.07 (15.31)	12 (5.4)	Self‐employed	15 (6.7)	
Number of pain locations (*n*)	3.45 (2.24)	16 (7.2)	White‐collar worker	29 (13.0)	
PPT m. tibialis anterior (Newton)	50.89 (24.81)	3 (1.3)	Labourer	26 (11.7)	
PPT medial knee (Newton)	42.83 (23.71)	3 (1.3)	Unemployed	2 (0.9)	
PPT lateral knee (Newton)	48.06 (26.58)	3 (1.3)	Other	26 (11.7)	
PPT m. ECRL (Newton)	37.55 (17.24)	3 (1.3)	Education	12 (5.4)	11 (4.9)
PPT forehead (Newton)	30.18 (12.73)	38 (17)	No degree	12 (5.4)	
TS after sensation medial knee (0–10)	0.40 (1.11)	4 (1.8)	Primary	47 (21.1)	
TS medial knee (difference in NRS)	1.23 (2.02)	3 (1.3)	Technical secondary	1 (0.4)	
TS after sensation medial wrist (0–10)	0.16 (0.59)	4 (1.8)	Higher secondary	50 (22.4)	
TS medial wrist (difference in NRS)	0.98 (1.56)	4 (1.8)	High school	20 (9.0)	
TCA medial knee (0–10)	0.36 (0.96)	4 (1.8)	University	41 (18.4)	
THA medial knee (0–10)	0.82 (1.46)	4 (1.8)	Other	12 (5.4)	
TCA lateral knee (0–10)	0.27 (0.91)	4 (1.8)			
THA lateral knee (0–10)	0.37 (1.09)	4 (1.8)			
TCA m. ECRL (0–10)	0.19 (0.75)	4 (1.8)			
THA m. ECRL (0–10)	0.45 (1.11)	4 (1.8)			
CPM (%)	9.94 (48.31)	24 (10.8)			
CSI (0–100)	28.06 (13.14)	12 (5.4)			
Psychological variables					
PCS total score (0–52)	16.24 (10.33)	11 (4.9)			
HADS depression (0–21)	5.06 (3.26)	10 (4.5)			
HADS fear (0–21)	5.34 (4.01)	10 (4.5)			
KSSS expectation (3–15)	13.96 (1.63)	13 (5.8)			
KSSS satisfaction (0–40)	15.67 (7.35)	13 (5.8)			
IPQR timeline (6–30)	17.77 (5.25)	10 (4.5)			
IPQR consequences (6–30)	19.34 (4.21)	10 (4.5)			
IPQR timeline cyclical (4–25)	11.97 (3.85)	10 (4.5)			
IPQR personal control (6‐30)	19.74 (3.94)	10 (4.5)			
IPQR treatment control (5–25)	18.06 (3.10)	10 (4.5)			
IPQR emotional representations (6–30)	15.73 (4.63)	10 (4.5)			
IPQR illness coherence (5–25)	18.74 (2.12)	10 (4.5)			
IPQR identity (0–14)	2.07 (1.43)	9 (4)			
Outcome variables					
ΔKOOS subscale pain (diff KOOS FU‐BL)	28.66 (26.01)	60 (26.9)			
KOOS subscale pain FU (0–100)	73.45 (24.15)	55 (24.7)			

Abbreviations: BL, baseline; BMI, body mass index; CPM, conditioned pain modulation; CSI, Central Sensitisation Inventory; Diff, difference; ECRL, extensor carpi radialis longus; FU, follow‐up 1‐year post‐TKA; HADS, Hospitality Anxiety and Depression Scale; HbA1c, glycated haemoglobin; IPQR, Illness Perceptions Questionnaire Revised; kgf, kilograms force; K&L, Kellgren and Lawrence scale; KOOS, Knee Injury and Osteoarthritis Outcome Scale; KSSS, Knee Society Scoring System; m., musculus; NRS, numeric rating scale; PCS, pain catastrophising scale; PPT, pressure pain threshold; TCA, thermal cold allodynia; THA, thermal heat allodynia; TS, temporal summation; ΔKOOS, difference in KOOS score preoperative versus 1‐year postoperative.

### Missing data in the outcome variables and potential preoperative predictors

**Figure 1:**
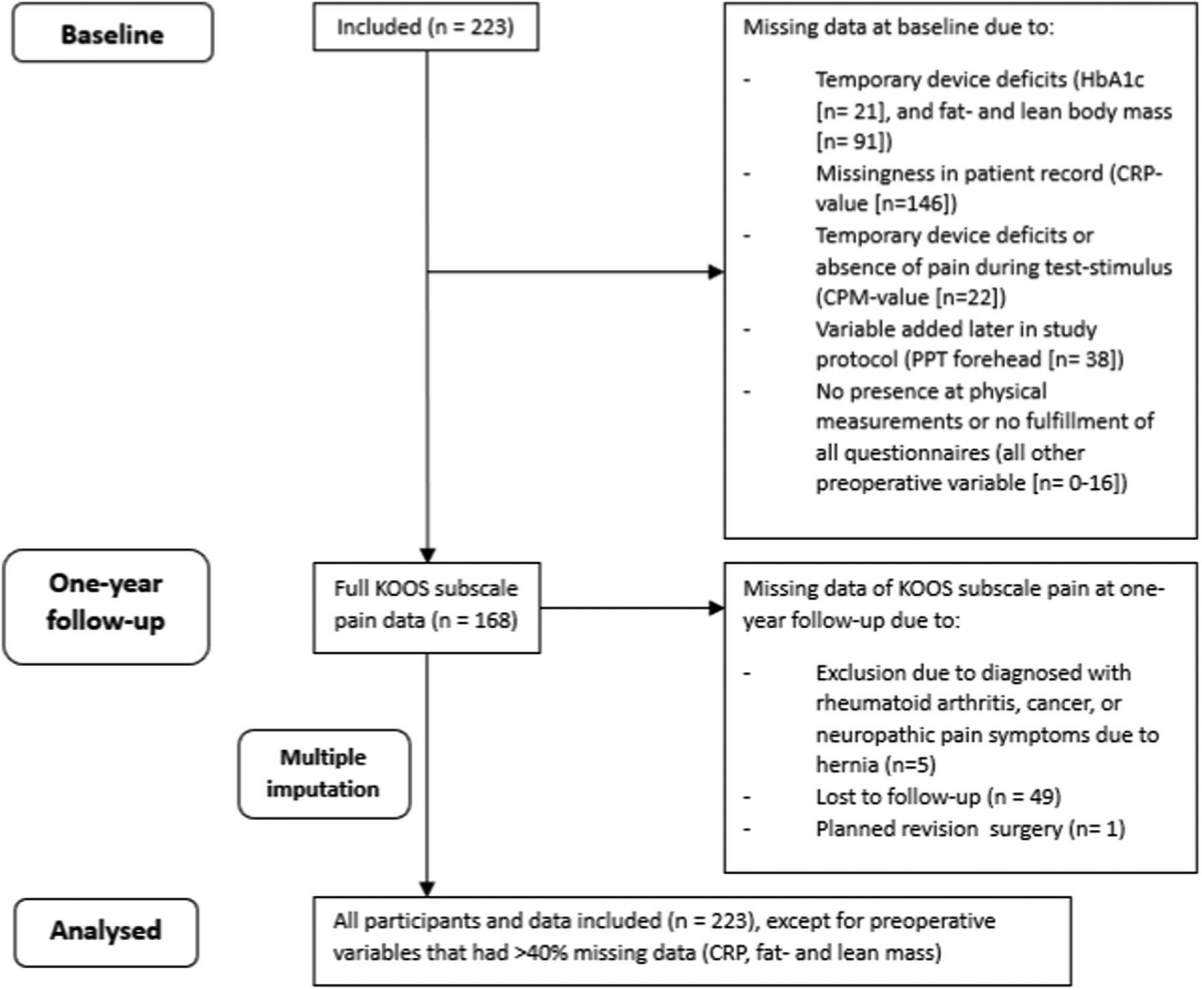
Flow diagram of missing data. CPM, conditioned pain modulation; CRP, creatine phosphate; HbA1c, glycated haemoglobin; KOOS, Knee Injury and Osteoarthritis Outcome Score; n, number of participants; PPT, pressure pain threshold.

A detailed overview of all missing data with reasons at baseline and 1‐year follow‐up can be found in Figure 1 and Table 3. The variables CRP value and fat and lean body mass were not imputed because the missingness was >40% [23]. These variables were therefore excluded from the analyses. However, for all other data, multiple imputation was used, and therefore all participants (*n* = 223) were analysed for all univariate correlation and both multivariable linear regression analyses.

### Univariate associations

#### Correlation between all possible predictors

To meet the assumption of nonmulticollinearity, the PPT measured at lateral knee‐joint line, m. Tibialis anterior and forehead, and thermal allodynia measured at lateral knee‐joint line were excluded for further analyses (high correlation [*r* > 0.70] with other possible predictors; Supporting Information S1: Resource 1).

#### Correlation between each possible predictor and absolute KOOS subscale pain scores 1‐year post‐TKA, on the one hand, and ΔKOOS subscale pain, on the other hand

Seventeen variables were associated with the *KOOS subscale pain score 1 year post‐TKA*, and 14 variables with the *ΔKOOS subscale pain*, each with a *p* value <0.2. These were consequently included at the start of the multivariable regression model (Table 4).

**Table 4:** Results of univariate associations.

	KOOS subscale pain FU	ΔKOOS subscale pain
Predictors	*r* Value (*p* Value)	
Demographic variables		
Age	0.138 (0.073)*	0.014 (0.853)
Sex	0.037 (0.600)	0.004 (0.953)
Metabolic and inflammatory variables		
BMI	0.057 (0.432)	0.106 (0.130)*
HbA1c	−0.210 (0.008)*	−0.186 (0.016)*
Functional variables		
Strength m. Quadriceps	0.066 (0.386)	−0.054 (0.488)
Strength m. Hamstrings	0.028 (0.723)	−0.070 (0.389)
Proprioception	0.037 (0.612)	−0.015 (0.835)
KOOS symptoms	−0.022 (0.774)	−0.170 (0.026)*
30CST	0.054 (0.507)	−0.029 (0.719)
KSSS functional score	0.088 (0.229)	−0.296 (<0.001)*
Pain‐related variables		
KOOS subscale pain BL	0.189 (0.011)*	0.397 (<0.001)*
Number of pain locations	−0.270 (<0.001)*	−0.136 (0.075)*
PPT m. Tibialis anterior	/	/
PPT medial knee	0.101 (0.198)*	−0.005 (0.951)
PPT lateral knee	/	/
PPT m. ECRL	0.151 (0.049)*	0.017 (0.855)
PPT forehead	/	/
TS after sensation medial knee	−0.079 (0.317)	−0.047 (0.557)
TS medial knee	−0.03 (0.690)	0.028 (0.719)
TS after sensation medial wrist	0.051 (0.470)	0.028 (0.684)
TS medial wrist	−0.026 (0.733)	0.023 (0.768)
TCA medial knee	−0.089 (0.298)	0.020 (0.803)
THA medial knee	−0.147 (0.047)*	−0.082 (0.273)
TCA lateral knee	/	/
THA lateral knee	/	/
TCA m. ECRL	−0.048 (0.541)	0.018 (0.811)
THA m. ECRL	−0.108 (0.174)*	−0.054 (0.487)
CPM	0.077 (0.346)	−0.054 (0.488)
CSI	−0.328 (<0.001)*	−0.172 (0.022*)
Psychological variables		
PCS total score	−0.159 (0.035)*	−0.007 (0.920)
HADS depression	−0.054 (0.504)	0.025 (0.744)
HADS anxiety	−0.189 (0.030)*	−0.119 (0.134)*
KSSS expectation	0.121 (0.119)*	0.091 (0.247)
KSSS satisfaction	0.292 (<0.001)*	−0.124 (0.124)*
IPQR timeline	−0.012 (0.882)	0.030 (0.683)
IPQR consequences	−0.066 (0.349)	0.084 (0.277)
IPQR timeline cyclical	−0.074 (0.333)	−0.124 (0.107)*
IPQR personal control	−0.154 (0.052)*	−0.246 (0.002)*
IPQR treatment control	−0.076 (0.284)	−0.161 (0.020)*
IPQR emotional representations	−0.179 (0.017)*	−0.034 (0.643)
IPQR illness coherence	0.034 (0.670)	−0.002 (0.976)
IPQR identity	−0.155 (0.044)*	−0.043 (0.568)
Structural variables		
K&L scale	0.211 (0.010)*	0.108 (0.181)*
Social variables		
Marital status	−0.084 (0.311)	−0.101 (0.230)
Work	−0.004 (0.953)	0.109 (0.140)*
Education	−0.029 (0.720)	−0.032 (0.685)

Abbreviations: BL, baseline; BMI, body mass index; CPM, conditioned pain modulation; CSI, Central Sensitisation Inventory; Diff, difference; ECRL, extensor carpi radialis longus; FU, follow‐up 1‐year post‐TKA; HADS, Hospitality Anxiety and Depression Scale; HbA1c, glycated haemoglobin; IPQR, Illness Perceptions Questionnaire Revised; K&L, Kellgren and Lawrence scale; KOOS, Knee Injury and Osteoarthritis Outcome Scale; KSSS, Knee Society Scoring System; m., musculus; NRS, numeric rating scale; PCS, pain catastrophising scale; PPT, pressure pain threshold; TCA, thermal cold allodynia; THA, thermal heat allodynia; TS, temporal summation; ΔKOOS, difference in KOOS score preoperative versus 1‐year postoperative.

* *p* Value < 0.2.

### Multivariable regression models

#### Data preparation

The variance inflation factor indicated no multicollinearity. The linearity assumption was not met for PPT measured at medial knee‐joint line, the Knee Society Scoring System (KSSS) subscale expectation and the Illness Perceptions Questionnaire Revised (IPQR) subscale treatment control and heat allodynia measured at m. Extensor Carpi Radialis Longus (ECRL). Therefore, these variables were transformed into their logarithmic value, except for heat allodynia, for which a Box–Cox transformation was used. In addition, the linearity assumption was not met for the Central Sensitisation Inventory (CSI) and BMI, but these were transformed to categorical variables (CSI: 1 = CSI score ≥40 and 0 = CSI score <40 [31]; BMI: 0 = <25 kg/cm^2^, 1 = 25–29.9 kg/cm^2^, 2 = ≥30 kg/cm^2^ [60]) because other transformations did not fulfil the linearity assumption.

#### Multivariable regression models

The final multivariable regression models of *KOOS subscale pain score 1‐year post‐ TKA* and the *ΔKOOS subscale pain* had an adjusted *R* ^2^ of 0.25 and 0.37, respectively.

*Higher HbA1c values, higher number of pain locations, higher IPQR subscale personal control scores, a lower KSSS subscale satisfaction score, KOA grade (K&L scale grade 2) and a score of ≥40 on the CSI* were significant predictors for lower scores on the *KOOS subscale pain 1‐year after surgery* after backward selection (Table 5).

**Table 5:** Multiple linear regression model for KOOS subscale pain 1‐year after surgery.

Full multiple linear regression model		
Predictor	Exp (*B*) (95% CI)	*p* Value
(Constant)	142.26 (84.90, 199.62)	<0.001*
Age	0.06 (−0.43, 0.55)	0.688
HbA1c	−6.36 (−12.14, −0.58)	0.021*
KOOS subscale pain baseline	−0.09 (−0.43, 0.25)	0.603
Number of pain locations	−1.69 (−3.42, 0.05)	0.025*
PPT medial knee	−4.63 (−12.31, 3.05)	0.188
PPT m. ECRL	0.13 (−0.18, 0.43)	0.352
THA medial knee	−2.08 (−5.61, 1.46)	0.224
THA m. ECRL	0.84 (−5.62, 7.30)	0.674
CSI ≥ 40	−11.02 (−22.09, 0.05)	0.035*
PCS	0.01 (−0.38, 0.39)	0.838
HADS subscale anxiety	0.23 (−1.10, 1.56)	0.593
KSSS subscale satisfaction	0.77 (0.05, 1.48)	0.008*
KSSS subscale expectations	−9.75 (−22.15, 2.65)	0.104
IPQR subscale identity	−0.36 (−2.92, 2.21)	0.579
IPQR subscale personal control	−0.97 (−1.82, −0.11)	0.016*
IPQR emotional representations	−0.10 (−1.09, 0.89)	0.648
KL scale = grade 1	−17.87 (−48.44, 12.69)	0.094
KL scale = grade 2	−9.21 (−18.95, 0.52)	0.030*
KL scale = grade 3	−0.42 (−8.27, 7.44)	0.814
*R* ^2^ = 0.31 and adjusted *R* ^2^ = 0.25		

Final multiple linear regression model after backward selection		
Predictor	Exp (*B*) (95% CI)	*p* Value
(Constant)	126.46 (92.25, 160.67)	<0.001*
HbA1c	−5.62 (−11.08, −0.16)	0.029*
Number of pain locations	−1.61 (−3.28, 0.05)	0.025*
CSI ≥ 40	−10.91 (−20.93, −0.89)	0.011*
KSSS subscale satisfaction	0.69 (0.21, 1.16)	0.002*
IPQR subscale personal control	−1.13 (−1.97, −0.30)	0.002*
K&L scale = grade 1	−20.47 (−50.28, 9.33)	0.060
K&L scale = grade 2	−9.60 (−19.02, −0.17)	0.018*
K&L scale = grade 3	−1.11 (−9.07, 6.85)	0.656
*R* ^2^ = 0.27 and adjusted *R* ^2^ = 0.25		

*Note*: K&L scale = grade 4, and CSI < 40 are reference categories.

Abbreviations: CI, confidence interval; CSI, Central Sensitisation Inventory; ECRL, extensor carpi radialis longus; Exp (*B*), regression coefficient; HADS, Hospitality Anxiety and Depression Scale; HbA1c, glycated haemoglobin; IPQR, Illness Perceptions Questionnaire Revised; KOOS, Knee Injury and Osteoarthritis Outcome Scale; K&L, Kellgren and Lawrence scale; KSSS, Knee Society Scoring System; m., musculus; PCS, pain catastrophising scale; PPT, pressure pain threshold; THA, thermal heat allodynia.

* *p* Value < 0.05.

The *same variables* were significant predictors for negative or closer to zero *ΔKOOS subscale pain scores*; however, *K&L scale grade 1*, instead of K&L scale grade 2 was a significant predictor. Moreover, also a *higher KSSS subscale functional score, higher KOOS subscale pain score at baseline and work status* (being self‐employed after backward selection (Table 6).

**Table 6:** Multiple linear regression model for ΔKOOS subscale pain.

Full multiple linear regression model		
Predictor	Exp (*B*) (95% CI)	*p* Value
(Constant)	168.83 (108.82, 228.84)	<0.001*
BMI = 25–29.9 kg/m^2^	2.61 (−6.77, 11.99)	0.577
BMI = ≥30 kg/m^2^	6.89 (−2.35, 16.13)	0.116
HbA1c	−6.40 (−11.98, −0.83)	0.010*
KOOS subscale symptoms	−0.18 (−0.37, 0.02)	0.042*
KSSS subscale functional score	−0.20 (−0.53, 0.12)	0.164
KOOS subscale pain baseline	−0.94 (−1.28, −0.60)	<0.001*
Number of pain locations	−1.81 (−3.50, −0.11)	0.011*
CSI ≥ 40	−12.98 (−23.94, −2.03)	0.006*
HADS subscale anxiety	0.19 (−0.86, 1.24)	0.591
KSSS subscale satisfaction	0.96 (0.24, 1.69)	0.002*
IPQR subscale treatment control	−8.21 (−25.90, 9.48)	0.270
IPQR subscale personal control	−0.91 (−1.79, −0.04)	0.021*
IPQR subscale timeline cyclical	−0.49 (−1.38, 0.40)	0.278
K&L scale = grade 1	−21.44 (−50.41, 7.53)	0.045*
K&L scale = grade 2	−7.73 (−17.35, 1.88)	0.074
K&L scale = grade 3	−1.08 (−8.78, 6.61)	0.623
Work = pension	−6.98 (−18.14, 4.17)	0.180
Work = self‐employed	−17.22 (−33.81, −0.62)	0.010*
Work = white‐collar worker	−4.03 (−18.82, 10.76)	0.586
Work = labourer	−12.67 (−26.59, 1.24)	0.042*
Work = unemployed	−14.30 (−59.01, 30.42)	0.358
*R* ^2^ = 0.44 and adjusted *R* ^2^ = 0.37		

Final multiple linear regression model after backward selection		
Predictor	Exp (*B*) (95% CI)	*p* Value
(Constant)	139.95 (104.58, 175.32)	<0.001*
HbA1c	−5.83 (−11.19, −0.47)	0.018*
KSSS subscale functional score	−0.29 (−0.60, 0.02)	0.022*
KOOS subscale pain baseline	−0.93 (−1.27, −0.59)	<0.001*
Number of pain locations	−1.71 (−3.37, −0.05)	0.014*
CSI ≥ 40	−11.71 (−21.91, −1.52)	0.006*
KSSS subscale satisfaction	0.91 (0.20, 1.63)	0.005*
IPQR subscale personal control	−1.06 (−1.92, −0.21)	0.009*
K&L scale = grade 1	−23.29 (−52.55, 5.98)	0.033*
K&L scale = grade 2	−7.93 (−17.32, 1.47)	0.052
K&L scale = grade 3	−0.81 (−8.68, 7.07)	0.732
Work = pension	−8.78 (−19.71, 2.15)	0.057
Work = self‐employed	−16.89 (−33.58, −0.19)	0.012*
Work = white‐collar worker	−4.23 (−18.78, 10.31)	0.649
Work = labourer	−11.41 (−25.25, 2.43)	0.077
Work = unemployed	−11.75 (−55.62, 32.12)	0.436
*R* ^2^ = 0.41 and adjusted *R* ^2^ = 0.37		

*Note*: K&L scale = grade 4, CSI < 40, BMI < 25 kg/m^2^ and work = ‘other category’ are the reference categories.

Abbreviations: BMI, body mass index; CI, confidence interval; CSI, Central Sensitisation Inventory; Exp (*B*), regression coefficient; HADS, Hospitality Anxiety and Depression Scale; HbA1c, glycated haemoglobin; IPQR, Illness Perceptions Questionnaire Revised; K&L, Kellgren and Lawrence scale; KOOS, Knee Injury and Osteoarthritis Outcome Scale; KSSS, Knee Society Scoring System; ΔKOOS, difference in KOOS score preoperative versus 1‐year postoperative.

* *p* Value < 0.05.

All other variables were no significant predictors for both outcomes (*p* > 0.05). To ensure adequate interpretation of Tables 5 and 6, a real‐life example is presented in Table 7 to predict both outcomes.

**Table 7:** Example for the prediction of the KOOS subscale pain score 1‐year postoperative and ΔKOOS subscale pain score (after backward selection).

KOOS subscale pain score 1‐year postoperative	
*Data of patient (example)*: HbA1c value: 5.7 Number of pain locations: 3 CSI ≥ 40: Yes KSSS subscale satisfaction score: 10 IPQR subscale personal control score: 16 K&L scale: 2	*KOOS subscale pain score 1‐year postoperative* = 126.46 − (5.62 × 5.7) − (1.61 × 3) − (10.91 × 1) + (0.69 × 10) − (1.13 × 16) − (20.47 × 0) − (9.60 × 1) − (1.11 × 0) = 57.91
ΔKOOS subscale pain score	
*Data of patient (example)*: HbA1c: 5.7 KSSS subscale functional score: 30 KOOS subscale pain baseline score: 55 Number of pain locations: 3 CSI ≥ 40: Yes KSSS subscale satisfaction: 10 IPQR subscale personal control: 16 K&L scale: 2 Work: Unemployed	*ΔKOOS subscale pain score* = 139.95 − (5.83 × 5.7) − (0.29 × 30) − (0.93 × 55) − (1.71 × 3) − (11.71 × 1) + (0.91 × 10) − (1.06 × 16) − (23.29 × 0) − (7.93 × 1) − (0.81 × 0) − (8.78 × 0) − (16.89 × 0) − (4.23 × 0) − (11.41 × 0) − (11.75 × 1) = 2.49

Abbreviations: CSI, Central Sensitisation Inventory; HbA1c, glycated haemoglobin; IPQR, Illness Perceptions Questionnaire Revised; K&L, Kellgren and Lawrence scale; KOOS, Knee Injury and Osteoarthritis Outcome Scale; KSSS, Knee Society Scoring System; ΔKOOS, difference in KOOS score preoperative versus 1‐year postoperative.

## DISCUSSION

The most important findings of the current study were the following: higher HbA1c values and number of pain locations, lower preoperative satisfaction, KOA grade and personal control, and self‐reported symptoms of central sensitisation were consistent preoperative predictors for both more pain and pain deterioration or less pain improvement 1‐year post‐TKA. In addition, also being self‐employed, less preoperative pain and better self‐reported function appeared to be predictors for pain deterioration or less pain improvement 1‐year post‐TKA. The multivariable regression model for 1‐year post‐TKA pain and pain deterioration or less pain improvement 1‐year post‐TKA had an adjusted *R* ^2^ of 0.25 and 0.37 after backward selection, respectively.

### Interpretation of results and relation to previous literature

*HbA1c* is a measure of glycemic control. Previous research has been inconclusive regarding the role of diabetes in chronic post‐TKA pain [2, 24, 40, 49]. However, these studies only measured self‐reported presence of diabetes, overlooking the nuanced assessment provided by HbA1c concentration (which goes broader than the presence of diabetes). Our study emphasises the importance of HbA1c levels in their potential predictive role for 1‐year post‐TKA pain when higher values (=less adequate blood sugar control in people with or without diabetes [48]) are present.

Furthermore, both widespread pain (*high number of pain locations*) and *self‐reported symptoms of central sensitisation* may be indicative of having disturbed somatosensory functioning [28, 31], which has been previously found to be predictive of chronic postoperative pain [11, 14, 16, 18]. Nevertheless, the current study showed that quantitative sensory testing (QST) was not predictive of post‐TKA pain. As reported in the systematic review of Paredes et al [36], the predicted role of QST parameters also remains unclear in previous research, mainly due to heterogeneous methodologies used across different studies.

To the best of our knowledge, *preoperative satisfaction* about knee pain during various functional activities was not previously examined as a possible predictor for poor TKA outcome. The current study showed that low preoperative satisfaction was an important predictor for more pain 1‐year post‐TKA, while the baseline pain intensity score was no predictor. Satisfaction about pain during functional activities is not only influenced by pain intensity itself but also by other factors (expectations, psychological factors, etc. [59]). Previous research indicated no consistent association between pain intensity and satisfaction [38], indicating the importance of measuring satisfaction on top of pain intensity.

*Minimal structural knee damage* being a predictor of post‐TKA pain aligns with findings of previous systematic reviews [12, 64]. This could be explained by the weak associations found between structural and clinical features [6], which is also typical for KOA patients presenting with disturbed somatosensory functioning [17] and can be indicative of chronic primary musculoskeletal pain (i.e., in which the pain is or has become a condition in its own right and not related to the musculoskeletal condition anymore [54]. These findings suggest considering delaying surgical interventions and prioritising alternative treatment strategies when low structural damage is present.

Although another study showed no association between self‐efficacy and chronic TKA pain [62], the current study showed that *better personal control* was predictive of worse post‐TKA pain. This was contrary to our expectations [3, 16]. However, an explanation could be that individuals with low ‘personal control’ had actually no other ‘personal control’ option than placing a TKA anymore to improve pain intensity. This is the first study to include the IPQR as a possible predictor of chronic post‐TKA pain, which makes comparison with other studies difficult.

Interestingly, *less pain intensity at baseline* and *better function* were also predictors (pain deterioration or less pain improvement). This could be attributed to a ceiling effect, implying individuals with only mild symptoms might perceive a narrower margin for pain intensity improvement. Conversely, those with more severe symptoms could have a wider margin for perceived improvement [55]. No correction for participants scoring more extreme scores was made in this study, indicating the need for further research into this factor's contribution to post‐TKA pain scores.

Last, *being self‐employed* was also predictive of pain deterioration or less pain improvement. This is the only social factor being predictive, while marital status or education levels were not. Being self‐employed often also means no or less income while on ‘sick leave’, which can be associated with more stress, which in turn is interrelated with chronic pain. Additionally, self‐employed individuals may return to work sooner and may not be able to devote sufficient attention to comprehensive rehabilitation [29]. Remarkably, Edwards et al. found that higher education and not the status of employment was predictive at the final multivariable prediction model to predict pain intensity 6 months post‐TKA [14].

Notably, baseline pain intensity score was not predictive for pain 1‐year post‐TKA, and anxiety and pain catastrophising were not predictive for both outcomes, which contrasts with previous research [14, 16, 18]. An explanation could be that better preoperative satisfaction filtered out the baseline pain intensity (almost strongly correlated; Supporting Information S1: Table 1), and that self‐reported symptoms of central sensitisation filtered out pain catastrophising and anxiety (moderately correlated; Supporting Information S1: Table 1). The CSI also measures several psychological constructs, and previous research even found strong correlation between pain catastrophising and anxiety [1]. In the current study, only variables having a variance inflation factor of >4 or correlated >0.7 with another possible predictor were excluded at the start of the multivariable regression model.

All multivariable models showed an (adjusted) *R* ^2^ of 0.26 or higher, indicative of an acceptable effect [35]. This suggests that a significant portion of the variance in pain 1‐year post‐TKA and pain deterioration or less pain improvement post‐TKA is explained by the predictors in these models. These findings align with those reported by Edwards et al., whose methodology was similar to ours and demonstrated an *R* ^2^ of 0.34 [14]. However, a significant portion (69% vs. 56%) remains unexplained, highlighting the importance of further research.

### Implications for future research and clinical practice

This study provides valuable information for future studies to select the most important potential predictors in foretelling the presence of chronic post‐TKA pain or ‘treatment success’ (decided based on absolute post‐TKA pain score or on reaching the minimal clinical important change when valid cut‐off points have been identified). However, more studies are needed that incorporate as many potential predictors of chronic‐TKA pain as possible in one linear multivariable regression model to identify the consistent and most important predictors and should focus on consistent and easy‐to‐use measures in clinical practice. This can ultimately lead to an internally and externally validated clinical risk assessment tool. Future prehabilitation research should then investigate if positively changing the modifiable identified factors (e.g. *self‐reported symptoms of central sensitisation, higher HbA1c, lower preoperative satisfaction, higher number of pain locations and better personal control* in the current study) with stratified treatment modalities would result in better postoperative outcomes [56]. For clinical practice, making the patients aware of possible negative predictors can provide valuable insights for shared decision‐making between the caregivers and the patient regarding the focus of the treatment and realistic expectations of TKA. This approach can increase patients' engagement in the treatment but also assists caregivers in offering more tailored and effective treatment [53].

## STRENGTHS AND LIMITATIONS

This was the first multicenter study to evaluate different possible predictors covering the entire biopsychosocial model using multivariable regression models with a follow‐up period of 1‐year post‐TKA. Thereupon, the presentation of both 1‐year post‐TKA pain, as well as pain deterioration or less pain improvement, and the large size effects of the predictors (acceptable *R* ^2^) enhance its value [35]. However, also limitations of the study need to be addressed. First, no a priori sample size calculation was performed, but full power (at least 10 subjects for each possible predictor [20]) was preserved by first selecting possible predictors using univariate associations. Second, linear regression to predict post‐TKA pain scores was used instead of logistic regression. As such, only absolute pain scores (higher or lower) or difference in pain scores (pain deterioration or less pain improvement) could be predicted and not the presence of chronic pain or not. However, this approach was chosen because no valid cut‐off points for the presence of chronic‐TKA pain or the minimal clinically important change of the KOOS subscale pain have been identified [42] and because dichotomising continuous variables includes the risk of losing (possible) important information. Thereupon, less potential predictors are allowed in logistic regression due to its dependence on the sample size of the smallest subgroup (i.e., ±20% are estimated to report chronic‐TKA pain [4, 44, 61]) [20]. Third, some participants rated the maximum temperature of the test stimulus for the conditioned pain modulation (CPM) measurement (46°C) lower than the originally pursued 4/10. Only participants scoring 0/10 were excluded from the analyses. It is possible that the test stimulus was not noxious enough in all participants, obscuring the real CPM effect. Fourth, missing data for fat and lean mass and CRP were very high (device deficits or not registered in the medical record) and could therefore not be analysed. Last, while our primary focus was on identifying preoperative predictors, it is important to note that perioperative and postoperative factors, which were not considered in this study, might also significantly influence postoperative outcomes [43].

## CONCLUSION

The study found that self‐reported symptoms of central sensitisation, higher HbA1c, satisfaction, less structural damage, higher number of pain locations and better personal control were consistent preoperative predictors of both more pain 1‐year post‐TKA and pain deterioration or less pain improvement post‐TKA. In addition, being self‐employed, more pain at baseline and better function were significant preoperative predictors for pain deterioration or less pain improvement post‐TKA. Current results may be valuable for future studies that want to develop risk assessment tools for the prediction of chronic post‐TKA pain.

## AUTHOR CONTRIBUTIONS

Sophie Vervullens, Lotte Meert, Prof. Dr. Mira Meeus, Prof. Dr. Isabel Baert and Prof. Dr. Rob J. E. M. Smeets conceptualised and designed the study protocol. Sophie Vervullens and Lotte Meert were responsible for collection, analyses and interpretation of the data and wrote the draft of the article. Prof. Dr. Mira Meeus, Prof. Dr. Rob J. E. M. Smeets and Dr. Jonas Verbrugghe critically revised the analyses and drafts of the article. Prof. Dr. Peter Verdonk, Prof. Dr. Christiaan H. W. Heusdens and Dr. Frank Th. Rahusen provided the participants for the study and critically revised the draft of the article.

## CONFLICT OF INTEREST STATEMENT

The authors declare no conflict of interest.

## ETHICS STATEMENT

The ethical committees of both countries approved the study: BE300201319366 (Belgium) and NL6465408618 (the Netherlands). Informed consent was obtained from all participants.

## Supporting information

Supporting information.

Supporting information.

## Data Availability

The data sets generated during and/or analysed during the current study are available from the corresponding author upon reasonable request.
