# Supplementary material for: Preoperative glycaemic control, number of pain locations, structural knee damage, self‐reported central sensitisation, satisfaction and personal control are predictive of 1‐year postoperative pain, and change in pain from pre‐ to 1‐year posttotal knee arthroplasty
Source: Knee Surg Sports Traumatol Arthrosc. 2024 May 15;33(1):201–19. doi: 10.1002/ksa.12265 (PMC11716348; doi:10.1002/ksa.12265)
Supplement: Supplementary file 1 — Supporting information. [file KSA-33-201-s002.pdf]

Supplementary Table S1: correlation coefficients between predictors part 1

	Age	Education	BMI	**PPT m. TA**	**PPT mk**	**PPT lk**	**PPT m. ECRL**	**PPT forehead**	TS mk	TS mw	CPM	Strength m. Quadriceps	Strength m. Hamstrings	Proprio-ception	30s CST	Hb1Ac
Age	1	0,086	-0,144	0,095	0,025	0,033	0,031	0,118	-0,049	0,004	-0,068	-0,125	-0,071	0,145	-0,06	0,042
Education	0,086	1	0,018	-0,087	-0,127	-0,078	-0,011	-0,059	-0,021	0,015	-0,117	-0,013	-0,058	-0,192	0,13	0,116
BMI	-0,144	0,018	1	-0,108	-0,054	-0,055	0,06	-0,077	0,055	-0,053	0,093	0,111	0,113	-0,024	-0,2	0,161
**PPT m. TA**	0,095	-0,087	-0,108	1	**0,796**	**0,805**	**0,726**	0,656	-0,35	-0,223	-0,066	0,342	0,329	-0,033	0,208	-0,085
**PPT mk**	0,025	-0,127	-0,054	**0,796**	1	**0,764**	0,652	0,597	-0,306	-0,171	-0,131	0,343	0,316	-0,026	0,303	-0,146
**PPT lk**	0,033	-0,078	-0,055	**0,805**	**0,764**	1	**0,721**	0,694	-0,313	-0,27	-0,017	0,413	0,428	-0,017	0,23	-0,05
**PPT m. ECRL**	0,031	-0,011	0,06	**0,726**	0,652	**0,721**	1	**0,728**	-0,326	-0,291	-0,043	0,345	0,327	-0,028	0,147	-0,051
**PPT forehead**	0,118	-0,059	-0,077	0,656	0,597	0,694	**0,728**	1	-0,318	-0,287	-0,01	0,258	0,433	-0,098	0,222	0,072
TS mk	-0,049	-0,021	0,055	-0,35	-0,306	-0,313	-0,326	-0,318	1	0,418	0,064	-0,162	-0,215	0,045	-0,086	0,045
TS mw	0,004	0,015	-0,053	-0,223	-0,171	-0,27	-0,291	-0,287	0,418	1	-0,017	-0,203	-0,205	-0,032	-0,139	0,015
CPM	-0,068	-0,117	0,093	-0,066	-0,131	-0,017	-0,043	-0,01	0,064	-0,017	1	-0,048	-0,022	-0,05	-0,124	-0,004
Strength m. Quadriceps	-0,125	-0,013	0,111	0,342	0,343	0,413	0,345	0,258	-0,162	-0,203	-0,048	1	0,685	-0,029	0,461	0,009
Strength m. Hamstrings	-0,071	-0,058	0,113	0,329	0,316	0,428	0,327	0,433	-0,215	-0,205	-0,022	0,685	1	-0,109	0,371	-0,001
Proprioception	0,145	-0,192	-0,024	-0,033	-0,026	-0,017	-0,028	-0,098	0,045	-0,032	-0,05	-0,029	-0,109	1	-0,013	-0,033
30s CST	-0,06	0,13	-0,2	0,208	0,303	0,23	0,147	0,222	-0,086	-0,139	-0,124	0,461	0,371	-0,013	1	-0,018
Hb1Ac	0,042	0,116	0,161	-0,085	-0,146	-0,05	-0,051	0,072	0,045	0,015	-0,004	0,009	-0,001	-0,033	-0,018	1
Number of pain locations	-0,157	-0,065	0,08	-0,196	-0,196	-0,172	-0,181	-0,169	0,11	0,126	0,071	-0,189	-0,174	-0,047	-0,18	0,03
IPQR Identity	-0,079	0,066	0,147	-0,099	-0,152	-0,178	-0,103	-0,064	0,041	0,079	-0,094	-0,234	-0,128	-0,154	-0,037	0,031
IPQR timeline	-0,068	-0,008	-0,07	0,048	-0,004	0,019	-0,035	-0,051	0,118	0,052	-0,026	0,064	0,018	-0,078	-0,064	-0,106
IPQR consequences	-0,175	-0,105	0,109	-0,002	-0,05	-0,004	0,058	0,003	0,02	0,048	-0,083	0,014	-0,064	-0,051	-0,143	-0,068
IPQR personal control	-0,071	-0,016	0,046	0,075	0,111	0,036	0,043	0,024	-0,131	-0,026	-0,136	0,161	0,088	0,069	0,134	-0,025
IPQR treatment control	0,037	-0,032	-0,137	0,038	0,042	-0,01	-0,028	0,006	-0,099	0,044	-0,105	0,034	0,057	0,165	0,108	-0,05
IPQR illness coherence	-0,077	-0,015	-0,058	-0,07	-0,095	-0,02	-0,132	-0,068	0,035	0,071	-0,118	0,091	0,081	0,011	0,176	-0,023
IPQ timeline cyclical	0,049	-0,054	-0,048	0,033	0,059	-0,01	0,013	-0,04	-0,059	-0,128	-0,074	-0,007	-0,061	0,107	0,001	-0,063

IPQR emotional representations	-0,179	-0,049	0,259	-0,121	-0,154	-0,149	0,029	-0,053	0,104	0,075	0,017	-0,135	-0,145	-0,098	-0,196	0,021
PCS	-0,136	-0,187	0,199	-0,092	-0,098	-0,083	-0,05	-0,079	0,116	0,127	0,061	-0,069	-0,143	0,002	-0,128	0,101
HADS anxiety	-0,097	-0,135	0,075	-0,208	-0,141	-0,163	-0,101	-0,096	0,096	0,119	0,042	-0,16	-0,181	0,005	-0,223	0,023
HADS depression	-0,095	-0,13	0,113	-0,028	-0,017	0,047	0,062	0,046	0,059	0,035	-0,037	-0,006	-0,087	-0,037	-0,233	0,047
KSSS symptoms	0,256	0,056	-0,093	0,06	0,023	0,039	0,047	-0,045	-0,043	-0,01	-0,144	0,083	0,02	0,047	0,11	0,073
KSSS satisfaction	0,202	-0,039	-0,162	0,175	0,139	0,193	0,149	0,082	-0,085	-0,024	-0,022	0,18	0,156	0,097	0,099	-0,019
KSSS functional score	0,072	0,022	-0,218	0,159	0,197	0,236	0,164	0,145	-0,173	-0,106	-0,013	0,249	0,237	0,115	0,28	-0,078
KOOS symptoms	0,248	0,077	0,047	0,027	0,003	0,044	0,086	0,041	-0,096	-0,126	0,019	0,071	0,137	0,149	0,067	0,037
CSI	-0,195	0,015	0,122	-0,222	-0,259	-0,176	-0,227	-0,195	0,182	0,136	-0,055	-0,208	-0,197	-0,07	-0,21	0,182
KOOS subscale pain	0,202	0,029	-0,093	0,134	0,172	0,175	0,211	0,13	-0,098	-0,082	0,011	0,201	0,167	0,086	0,138	-0,019
K-L scale	0,056	-0,024	-0,002	0,141	0,197	0,191	0,158	0,106	-0,092	0,063	-0,027	0,189	0,152	-0,09	0,173	-0,052
Marital status	-0,058	-0,066	-0,029	-0,089	-0,054	-0,042	-0,124	-0,124	0,11	0,075	0,055	-0,073	-0,115	0,064	-0,005	-0,035
Work	-0,662	0,018	0,213	-0,107	-0,086	-0,082	-0,052	-0,146	0,011	0,002	0,023	0,103	0,039	-0,091	-0,01	-0,072
TS after sens mk	-0,114	0,032	-0,003	-0,24	-0,224	-0,269	-0,224	-0,207	0,356	0,141	-0,046	-0,211	-0,274	0,032	-0,109	0,014
TS after sens mw	-0,149	-0,02	-0,106	-0,058	-0,03	-0,134	-0,129	-0,127	0,148	0,26	-0,052	-0,077	-0,17	0,03	-0,055	-0,109
TH cold mk	-0,139	-0,048	-0,053	-0,175	-0,239	-0,271	-0,205	-0,277	0,166	0,103	-0,019	-0,148	-0,234	0,033	-0,123	-0,01
TH heat mk	-0,014	-0,033	-0,022	-0,189	-0,221	-0,276	-0,192	-0,219	0,118	0,119	-0,066	-0,091	-0,178	-0,049	-0,042	0,023
TH cold lk	-0,143	-0,07	0,004	-0,213	-0,209	-0,265	-0,254	-0,311	0,209	0,188	-0,092	-0,133	-0,186	-0,014	-0,115	-0,042
TH heat lk	-0,057	-0,1	0,033	-0,237	-0,23	-0,299	-0,261	-0,291	0,157	0,15	-0,051	-0,06	-0,217	-0,098	-0,102	-0,012
TH cold m. ECRL	-0,088	-0,009	-0,092	-0,148	-0,152	-0,232	-0,214	-0,293	0,098	0,12	-0,154	-0,048	-0,125	0	-0,069	-0,087
TH heat m. ECRL	0,036	-0,06	-0,093	-0,091	-0,106	-0,169	-0,176	-0,243	0,061	0,074	-0,156	-0,031	-0,18	0,019	-0,004	-0,017
KSSS expectations	0,032	-0,07	0,051	0,001	-0,015	0,004	-0,031	-0,113	-0,054	-0,005	0,008	0,044	0,008	0,128	0,064	-0,065
Sex	0,042	-0,16	-0,041	0,384	0,271	0,353	0,311	0,248	-0,259	-0,111	-0,069	0,556	0,443	0,011	0,147	-0,079

Abbreviations: 30CST = 30 seconds chair stand test, BMI = body mass index, CPM = conditioned pain modulation, CSI = Central Sensitization Index, ECRL = m. Extensor Carpi Radialis Longus, HADS = Hospital Anxiety and Depression Scale, Hb1Ac = glycated hemoglobin, IPQR = illness perceptions questionnaire revised, KSSS = Knee Society Scoring System, KOOS = Knee Injury and Osteoarthritis Outcome Score, lk = lateral knee, mk = medial knee, mw = medial wrist, m. = musculus, PCS = Pain Catastrophizing Scale, PPT = pressure pain threshold, TA = m. Tibialis Anterior, TH = thermal hypersensitivity, TS = temporal summation

Supplementary Table S2: correlation coefficients between predictors part 2

	Number of pain locations	IPQR Identity	IPQR timeline	IPQR consequences	IPQR personal control	IPQR treatment control	IPQR illness coherence	IPQ timeline cyclical	IPQR emotional representations	PCS	HADS anxiety	HADS depression	KSSS symptoms	KSSS satisfaction	KSSS functional score
Age	-0,157	-0,079	-0,068	-0,175	-0,071	0,037	-0,077	0,049	-0,179	-0,136	-0,097	-0,095	0,256	0,202	0,072
Education	-0,065	0,066	-0,008	-0,105	-0,016	-0,032	-0,015	-0,054	-0,049	-0,187	-0,135	-0,13	0,056	-0,039	0,022
BMI	0,08	0,147	-0,07	0,109	0,046	-0,137	-0,058	-0,048	0,259	0,199	0,075	0,113	-0,093	-0,162	-0,218
PPT m. TA	-0,196	-0,099	0,048	-0,002	0,075	0,038	-0,07	0,033	-0,121	-0,092	-0,208	-0,028	0,06	0,175	0,159
PPT mk	-0,196	-0,152	-0,004	-0,05	0,111	0,042	-0,095	0,059	-0,154	-0,098	-0,141	-0,017	0,023	0,139	0,197
PPT lk	-0,172	-0,178	0,019	-0,004	0,036	-0,01	-0,02	-0,01	-0,149	-0,083	-0,163	0,047	0,039	0,193	0,236
PPT m. ECRL	-0,181	-0,103	-0,035	0,058	0,043	-0,028	-0,132	0,013	0,029	-0,05	-0,101	0,062	0,047	0,149	0,164
PPT forehead	-0,169	-0,064	-0,051	0,003	0,024	0,006	-0,068	-0,04	-0,053	-0,079	-0,096	0,046	-0,045	0,082	0,145
TS mk	0,11	0,041	0,118	0,02	-0,131	-0,099	0,035	-0,059	0,104	0,116	0,096	0,059	-0,043	-0,085	-0,173
TS mw	0,126	0,079	0,052	0,048	-0,026	0,044	0,071	-0,128	0,075	0,127	0,119	0,035	-0,01	-0,024	-0,106
CPM	0,071	-0,094	-0,026	-0,083	-0,136	-0,105	-0,118	-0,074	0,017	0,061	0,042	-0,037	-0,144	-0,022	-0,013
Strength m. Quadriceps	-0,189	-0,234	0,064	0,014	0,161	0,034	0,091	-0,007	-0,135	-0,069	-0,16	-0,006	0,083	0,18	0,249
Strength m. Hamstrings	-0,174	-0,128	0,018	-0,064	0,088	0,057	0,081	-0,061	-0,145	-0,143	-0,181	-0,087	0,02	0,156	0,237
Proprioception	-0,047	-0,154	-0,078	-0,051	0,069	0,165	0,011	0,107	-0,098	0,002	0,005	-0,037	0,047	0,097	0,115
30s CST	-0,18	-0,037	-0,064	-0,143	0,134	0,108	0,176	0,001	-0,196	-0,128	-0,223	-0,233	0,11	0,099	0,28
Hb1Ac	0,03	0,031	-0,106	-0,068	-0,025	-0,05	-0,023	-0,063	0,021	0,101	0,023	0,047	0,073	-0,019	-0,078
Number of pain locations	1	0,041	0,081	0,031	0,016	-0,064	0,004	0,07	0,242	0,241	0,322	0,245	-0,101	-0,215	-0,185
IPQR Identity	0,041	1	0,02	0,25	0,075	-0,112	0,023	-0,044	0,274	0,145	0,162	0,127	-0,211	-0,265	-0,17
IPQR timeline	0,081	0,02	1	-0,14	-0,321	-0,009	0,005	0,136	0,115	0,133	0,144	-0,039	0,032	-0,03	-0,14
IPQR consequences	0,031	0,25	0,229	1	-0,036	-0,16	-0,045	-0,047	0,481	0,28	0,231	0,324	-0,255	-0,256	-0,323
IPQR personal control	0,016	0,075	-0,14	-0,036	1	0,306	0,106	0,241	0,006	0,018	0,052	-0,027	0,06	0,065	0,192
IPQR treatment control	-0,064	-0,112	-0,321	-0,16	0,306	1	0,154	0,052	-0,23	-0,141	-0,058	-0,186	0,092	0,09	0,27
IPQR illness coherence	0,004	0,023	-0,009	-0,045	0,106	0,154	1	-0,204	-0,147	-0,139	-0,154	-0,131	0,032	0,045	0,057

IPQ timeline cyclical	0,07	-0,044	0,005	-0,047	0,241	0,052	-0,204	1	0,097	0,071	0,188	0,157	0,152	0,087	0,079
IPQR emotional representations	0,242	0,274	0,136	0,481	0,006	-0,23	-0,147	0,097	1	0,527	0,612	0,456	-0,217	-0,263	-0,309
PCS	0,241	0,145	0,115	0,28	0,018	-0,141	-0,139	0,071	0,527	1	0,555	0,479	-0,2	-0,174	-0,281
HADS anxiety	0,322	0,162	0,133	0,231	0,052	-0,058	-0,154	0,188	0,612	0,555	1	0,61	-0,142	-0,147	-0,136
HADS depression	0,245	0,127	0,144	0,324	-0,027	-0,186	-0,131	0,157	0,456	0,479	0,61	1	-0,133	-0,127	-0,236
KSSS symptoms	-0,101	-0,211	-0,039	-0,255	0,06	0,092	0,032	0,152	-0,217	-0,2	-0,142	-0,133	1	0,605	0,444
KSSS satisfaction	-0,215	-0,265	0,032	-0,256	0,065	0,09	0,045	0,087	-0,263	-0,174	-0,147	-0,127	0,605	1	0,55
KSSS functional score	-0,185	-0,17	-0,03	-0,323	0,192	0,27	0,057	0,079	-0,309	-0,281	-0,136	-0,236	0,444	0,55	1
KOOS symptoms	-0,099	-0,173	-0,132	-0,121	0,018	-0,002	0,027	-0,019	-0,138	-0,046	-0,077	-0,054	0,232	0,205	0,189
CSI	0,459	0,318	0,15	0,234	0,066	-0,164	-0,039	0,065	0,456	0,411	0,588	0,489	-0,186	-0,277	-0,296
KOOS subscale pain	-0,202	-0,178	-0,072	-0,255	0,18	0,158	0,06	0,096	-0,231	-0,246	-0,1	-0,132	0,535	0,692	0,66
K-L scale	-0,028	-0,127	0,008	-0,087	-0,041	0,032	-0,163	0,027	-0,014	-0,135	-0,093	-0,136	-0,052	0,063	0,169
Marital status	0,009	-0,024	-0,089	0,025	-0,06	0,035	0,022	0,103	-0,1	-0,029	0,031	0,043	0,026	-0,034	0,019
Work	0,18	0,115	-0,066	0,041	0,159	0,021	-0,077	0,008	-0,19	0,145	0,057	-0,022	0,043	-0,212	-0,151
TS after sens mk	0,158	0,108	0,078	0,163	0,094	0,025	0,065	-0,006	-0,009	0,048	0,012	0,096	-0,002	-0,121	-0,106
TS after sens mw	0,042	-0,021	0,079	0,017	0,025	0,057	0,104	-0,078	0,047	0,006	0,008	-0,005	-0,164	0,02	0,075
TH cold mk	0,181	0,064	0,077	0,075	0,108	0,047	0,066	0,082	0,105	0,141	0,174	0,169	0,079	-0,098	-0,132
TH heat mk	0,083	0,104	0,084	0,071	0,152	0,072	0,019	0,08	-0,042	0,114	0,14	0,14	0,138	0,009	-0,108
TH cold lk	0,133	0,084	0,016	0,044	0,093	-0,009	0,034	0,082	0,047	0,053	0,161	0,093	0,083	-0,072	-0,142
TH heat lk	0,108	0,047	0,063	0,064	0,192	0,015	-0,059	0	-0,019	0,196	0,204	0,154	0,115	0,019	-0,102
TH cold m. ECRL	0,118	0,024	0,084	0,046	0,109	-0,014	0,019	0,04	0,079	0,068	0,082	0,098	0,045	0,031	-0,085
TH heat m. ECRL	0,042	0,017	0,082	0,023	0,09	-0,01	-0,012	0,063	0,005	0,045	0,042	0,049	0,106	0,023	-0,072
KSSS expectations	-0,085	-0,127	-0,258	-0,031	0,029	-0,009	0,147	0,07	-0,153	-0,037	-0,004	-0,127	-0,121	0,046	0,043
Sex	-0,16	-0,115	0,019	-0,162	0,084	0,049	0,074	0,114	-0,052	-0,102	-0,031	-0,211	-0,008	0,074	0,056

Abbreviations: 30CST = 30 seconds chair stand test, BMI = body mass index, CPM = conditioned pain modulation, CSI = Central Sensitization Index, ECRL = m. Extensor Carpi Radialis Longus, HADS = Hospital Anxiety and Depression Scale, Hb1Ac = glycated hemoglobin, IPQR = illness perceptions questionnaire revised, KSSS = Knee Society Scoring System, KOOS = Knee Injury and Osteoarthritis Outcome Score, lk = lateral knee, mk = medial knee, mw = medial wrist, m. = musculus, PCS = Pain Catastrophizing Scale, PPT = pressure pain threshold, TA = m. Tibialis Anterior, TH = thermal hypersensitivity, TS = temporal summation

Supplementary Table S3: correlation coefficients between predictors part 3

	KOOS symptoms	CSI	KOOS subscale pain	K-L scale	Marital status	Work	TS after sens mk	TS after sens mw	**TH cold mk**	**TH heat mk**	**TH cold lk**	**TH heat lk**	TH cold m. ECRL	TH heat m. ECRL	KSSS expectations	Sex
Age	0,248	-0,195	0,202	0,056	-0,058	-0,662	-0,114	-0,149	-0,139	-0,014	-0,143	-0,057	-0,088	0,036	0,032	0,042
Education	0,077	0,015	0,029	-0,024	-0,066	0,018	0,032	-0,02	-0,048	-0,033	-0,07	-0,1	-0,009	-0,06	-0,07	-0,16
BMI	0,047	0,122	-0,093	-0,002	-0,029	0,213	-0,003	-0,106	-0,053	-0,022	0,004	0,033	-0,092	-0,093	0,051	-0,041
PPT m. TA	0,027	-0,222	0,134	0,141	-0,089	-0,107	-0,24	-0,058	-0,175	-0,189	-0,213	-0,237	-0,148	-0,091	0,001	0,384
PPT mk	0,003	-0,259	0,172	0,197	-0,054	-0,086	-0,224	-0,03	-0,239	-0,221	-0,209	-0,23	-0,152	-0,106	-0,015	0,271
PPT lk	0,044	-0,176	0,175	0,191	-0,042	-0,082	-0,269	-0,134	-0,271	-0,276	-0,265	-0,299	-0,232	-0,169	0,004	0,353
PPT m. ECRL	0,086	-0,227	0,211	0,158	-0,124	-0,052	-0,224	-0,129	-0,205	-0,192	-0,254	-0,261	-0,214	-0,176	-0,031	0,311
PPT forehead	0,041	-0,195	0,13	0,106	-0,124	-0,146	-0,207	-0,127	-0,277	-0,219	-0,311	-0,291	-0,293	-0,243	-0,113	0,248
TS mk	-0,096	0,182	-0,098	-0,092	0,11	0,011	0,356	0,148	0,166	0,118	0,209	0,157	0,098	0,061	-0,054	-0,259
TS mw	-0,126	0,136	-0,082	0,063	0,075	0,002	0,141	0,26	0,103	0,119	0,188	0,15	0,12	0,074	-0,005	-0,111
CPM	0,019	-0,055	0,011	-0,027	0,055	0,023	-0,046	-0,052	-0,019	-0,066	-0,092	-0,051	-0,154	-0,156	0,008	-0,069
Strength m. Quadriceps	0,071	-0,208	0,201	0,189	-0,073	0,103	-0,211	-0,077	-0,148	-0,091	-0,133	-0,06	-0,048	-0,031	0,044	0,556
Strength m. Hamstrings	0,137	-0,197	0,167	0,152	-0,115	0,039	-0,274	-0,17	-0,234	-0,178	-0,186	-0,217	-0,125	-0,18	0,008	0,443
Proprioception	0,149	-0,07	0,086	-0,09	0,064	-0,091	0,032	0,03	0,033	-0,049	-0,014	-0,098	0	0,019	0,128	0,011
30s CST	0,067	-0,21	0,138	0,173	-0,005	-0,01	-0,109	-0,055	-0,123	-0,042	-0,115	-0,102	-0,069	-0,004	0,064	0,147
Hb1Ac	0,037	0,182	-0,019	-0,052	-0,035	-0,072	0,014	-0,109	-0,01	0,023	-0,042	-0,012	-0,087	-0,017	-0,065	-0,079
Number of pain locations	-0,099	0,459	-0,202	-0,028	0,009	0,18	0,158	0,042	0,181	0,083	0,133	0,108	0,118	0,042	-0,085	-0,16
IPQR Identity	-0,192	0,113	-0,508	-0,087	0,025	0,041	0,163	0,017	0,075	0,071	0,044	0,064	0,046	0,023	-0,031	-0.162
IPQR timeline	-0,132	0,15	-0,072	0,008	-0,089	-0,066	0,078	0,079	0,077	0,084	0,016	0,063	0,084	0,082	-0,258	0,019
IPQR consequences	-0,121	0,234	-0,255	-0,041	-0,06	0,159	0,094	0,025	0,108	0,152	0,093	0,192	0,109	0,09	0,029	0,084
IPQR personal control	0,018	0,066	0,18	0,032	0,035	0,021	0,025	0,057	0,047	0,072	-0,009	0,015	-0,014	-0,01	-0,009	0,049
IPQR treatment control	-0,002	-0,164	0,158	-0,163	0,022	-0,077	0,065	0,104	0,066	0,019	0,034	-0,059	0,019	-0,012	0,147	0,074
IPQR illness coherence	0,027	-0,039	0,06	0,027	0,103	0,008	-0,006	-0,078	0,082	0,08	0,082	0	0,04	0,063	0,07	0,114
IPQ timeline cyclical	-0,019	0,065	0,096	-0,014	-0,1	-0,19	-0,009	0,047	0,105	-0,042	0,047	-0,019	0,079	0,005	-0,153	-0,052

IPQR emotional representations	-0,138	0,456	-0,231	-0,135	-0,029	0,145	0,048	0,006	0,141	0,114	0,053	0,196	0,068	0,045	-0,037	-0,102
PCS	-0,046	0,411	-0,246	-0,093	0,031	0,057	0,012	0,008	0,174	0,14	0,161	0,204	0,082	0,042	-0,004	-0,031
HADS anxiety	-0,077	0,588	-0,1	-0,136	0,043	-0,022	0,096	-0,005	0,169	0,14	0,093	0,154	0,098	0,049	-0,127	-0,211
HADS depression	-0,054	0,489	-0,132	-0,052	0,026	0,043	-0,002	-0,164	0,079	0,138	0,083	0,115	0,045	0,106	-0,121	-0,008
KSSS symptoms	0,232	-0,186	0,535	0,063	-0,034	-0,212	-0,121	0,02	-0,098	0,009	-0,072	0,019	0,031	0,023	0,046	0,074
KSSS satisfaction	0,205	-0,277	0,692	0,169	0,019	-0,151	-0,106	0,075	-0,132	-0,108	-0,142	-0,102	-0,085	-0,072	0,043	0,056
KSSS functional score	0,189	-0,296	0,66	0,149	-0,082	-0,089	-0,025	0,13	-0,093	-0,1	-0,147	-0,115	-0,024	-0,032	0,024	0,191
KOOS symptoms	1	-0,138	0,261	0,172	-0,056	-0,127	-0,086	-0,083	-0,083	-0,001	-0,076	-0,073	-0,051	-0,024	0,008	0,136
CSI	-0,138	1	-0,234	-0,199	0,067	0,132	0,097	-0,022	0,1	0,124	0,071	0,174	0,103	0,092	-0,141	-0,27
KOOS subscale pain	0,261	-0,234	1	0,157	0,067	-0,212	-0,079	0,052	-0,152	-0,081	-0,23	-0,143	-0,092	-0,068	0,021	0,077
K-L scale	0,172	-0,199	0,157	1	-0,065	0,078	-0,106	0,02	-0,078	-0,103	-0,019	-0,104	-0,056	-0,072	0,031	0,174
Marital status	-0,056	0,067	0,067	-0,065	1	0,064	0,147	0,063	0,091	0,008	0,093	0,107	0,042	0,049	-0,005	-0,105
Work	-0,127	0,132	-0,212	0,078	0,064	1	0,089	0,058	0,125	-0,001	0,178	0,03	0,029	-0,097	0,032	-0,035
TS after sens mk	-0,086	0,097	-0,079	-0,106	0,147	0,089	1	0,384	0,366	0,204	0,254	0,188	0,155	0,149	0,021	-0,191
TS after sens mw	-0,083	-0,022	0,052	0,02	0,063	0,058	0,384	1	0,179	0,12	0,138	0,19	0,256	0,146	-0,01	-0,097
**TH cold mk**	-0,083	0,1	-0,152	-0,078	0,091	0,125	0,366	0,179	1	0,517	**0,708**	0,471	0,575	0,355	-0,097	-0,01
**TH heat mk**	-0,001	0,124	-0,081	-0,103	0,008	-0,001	0,204	0,12	0,517	1	0,395	**0,702**	0,391	0,66	-0,122	0,066
**TH cold lk**	-0,076	0,071	-0,23	-0,019	0,093	0,178	0,254	0,138	**0,708**	0,395	1	0,49	0,664	0,355	-0,038	-0,041
**TH heat lk**	-0,073	0,174	-0,143	-0,104	0,107	0,03	0,188	0,19	0,471	**0,702**	0,49	1	0,411	0,623	-0,041	0,053
TH cold m. ECRL	-0,051	0,103	-0,092	-0,056	0,042	0,029	0,155	0,256	0,575	0,391	0,664	0,411	1	0,475	-0,08	-0,005
TH heat m. ECRL	-0,024	0,092	-0,068	-0,072	0,049	-0,097	0,149	0,146	0,355	0,66	0,355	0,623	0,475	1	-0,125	0,075
KSSS expectations	0,008	-0,141	0,021	0,031	-0,005	0,032	0,021	-0,01	-0,097	-0,122	-0,038	-0,041	-0,08	-0,125	1	0,128
Sex	0,136	-0,27	0,077	0,174	-0,105	-0,035	-0,191	-0,097	-0,01	0,066	-0,041	0,053	-0,005	0,075	0,128	1

Abbreviations: 30CST = 30 seconds chair stand test, BMI = body mass index, CPM = conditioned pain modulation, CSI = Central Sensitization Index, ECRL = m. Extensor Carpi Radialis Longus, HADS = Hospital Anxiety and Depression Scale, Hb1Ac = glycated hemoglobin, IPQR = illness perceptions questionnaire revised, KSSS = Knee Society Scoring System, KOOS = Knee Injury and Osteoarthritis Outcome Score, lk = lateral knee, mk = medial knee, mw = medial wrist, m. = musculus, PCS = Pain Catastrophizing Scale, PPT = pressure pain threshold, TA = m. Tibialis Anterior, TH = thermal hypersensitivity, TS = temporal summation
