# Supplementary material for: Preoperative glycaemic control, number of pain locations, structural knee damage, self‐reported central sensitisation, satisfaction and personal control are predictive of 1‐year postoperative pain, and change in pain from pre‐ to 1‐year posttotal knee arthroplasty
Source: Knee Surg Sports Traumatol Arthrosc. 2024 May 15;33(1):201–19. doi: 10.1002/ksa.12265 (PMC11716348; doi:10.1002/ksa.12265)
Supplement: Supplementary file 2 — Supporting information. [file KSA-33-201-s001.pdf]

Online Resource 2: Supplementary references of Table 2

42. Baert IAC, Lluch E, Struyf T, Peeters G, Van Oosterwijck S, Tuynman J, et al. (2018). Inter- and intrarater reliability of two proprioception tests using clinical applicable measurement tools in subjects with and without knee osteoarthritis. Musculoskeletal Science and Practice.35:105-109.
43. Cathcart S, Winefield AH, Rolan P, Lushington K. (2009). Reliability of temporal summation and diffuse noxious inhibitory control. Pain Res Manag.14(6):433-438.
44. Collins NJ, Prinsen CA, Christensen R, Bartels EM, Terwee CB, Roos EM. (2016). Knee Injury and Osteoarthritis Outcome Score (KOOS): systematic review and meta-analysis of measurement properties. Osteoarthritis Cartilage.24(8):1317-1329.
45. Dams L, Haenen V, Van der Gucht E, Devoogdt N, Smeets A, Bernar K, et al. (2022). Absolute and Relative Reliability of a Comprehensive Quantitative Sensory Testing Protocol in Women Treated for Breast Cancer. Pain Med.23(6):1162-1175.
46. Dehghan M, Merchant AT. (2008). Is bioelectrical impedance accurate for use in large epidemiological studies? Nutr J.7:26.
47. Felson DT, Niu J, Guermazi A, Sack B, Aliabadi P. (2011). Defining radiographic incidence and progression of knee osteoarthritis: suggested modifications of the Kellgren and Lawrence scale. Annals of the rheumatic diseases.70(11):1884-1886.
48. Gill S, Hely R, Page RS, Hely A, Harrison B, Landers S. (2022). Thirty second chair stand test: Test–retest reliability, agreement and minimum detectable change in people with early-stage knee osteoarthritis. Physiotherapy Research International.27(3):e1957.
49. Hodge JM, Shah R, McCullough ML, Gapstur SM, Patel AV. (2020). Validation of self-reported height and weight in a large, nationwide cohort of U.S. adults. PLoS One.15(4):e0231229.
50. Hunter DJ, Zhang W, Conaghan PG, Hirko K, Menashe L, Reichmann WM, Losina E. (2011). Responsiveness and reliability of MRI in knee osteoarthritis: a meta-analysis of published evidence. Osteoarthritis and cartilage / OARS, Osteoarthritis Research Society.19(5):589-605.
51. Kregel J, Vuijk PJ, Descheemaeker F, Keizer D, van der Noord R, Nijs J, et al. (2016). The Dutch Central Sensitization Inventory (CSI): Factor Analysis, Discriminative Power, and Test-Retest Reliability. Clin J Pain.32(7):624-630.
52. Leysen M, Nijs J, Meeus M, Paul van Wilgen C, Struyf F, Vermandel A, et al. (2015). Clinimetric properties of illness perception questionnaire revised (IPQ-R) and brief illness perception questionnaire (Brief IPQ) in patients with musculoskeletal disorders: A systematic review. Manual Therapy.20(1):10-17.
53. Lipovšek T, Kacin A, Puh U. (2022). Reliability and validity of hand-held dynamometry for assessing lower limb muscle strength. Isokinetics and Exercise Science.30(3):231-240.
54. Mortensen JF, Kappel A, Rasmussen LE, Østgaard SE, Odgaard A. (2022). The Rosenberg view and coronal stress radiographs give similar measurements of articular cartilage height in knees with osteoarthritis. Archives of Orthopaedic and Trauma Surgery.142(9):2349-2360.
55. Noble PC, Scuderi GR, Brekke AC, Sikorskii A, Benjamin JB, Lonner JH, et al. (2012). Development of a new Knee Society scoring system. Clinical Orthopaedics and Related Research.470(1):20-32.
56. Ong WJ, Kwan YH, Lim ZY, Thumboo J, Yeo SJ, Yeo W, et al. (2021). Measurement properties of Pain Catastrophizing Scale in patients with knee osteoarthritis. Clinical Rheumatology.40(1):295-301.
57. Osman A, Barrios FX, Gutierrez PM, Kopper BA, Merrifield T, Grittmann L. (2000). The Pain Catastrophizing Scale: Further Psychometric Evaluation with Adult Samples. J Behav Med.23(4):351-365.
58. Park H-J, Kim SS, Lee S-Y, Park N-H, Park J-Y, Choi Y-J, Jeon H-J. (2013). A practical MRI grading system for osteoarthritis of the knee: association with Kellgren-Lawrence radiographic scores. Eur J Radiol.82(1):112-117.
59. PTS Diagnostics. A1CNow+ System 2023 [Available from: https://ptsdiagnostics.com/a1cnow-plus-system/.

60. Schiphof D, Klerk BMd, Koes BW, Bierma-Zeinstra S. (2008). Good reliability, questionable validity of 25 different classification criteria of knee osteoarthritis: a systematic appraisal. Journal of Clinical Epidemiology.61(12):1205-1215.e1202.
61. Schott GD. (2010). The cartography of pain: the evolving contribution of pain maps. European journal of pain (London, England).14(8):784-791.
62. Smith JW, Martins TB, Gopez E, Johnson T, Hill HR, Rosenberg TD. (2012). Significance of C-reactive protein in osteoarthritis and total knee arthroplasty outcomes. Ther Adv Musculoskelet Dis.4(5):315-325.
63. Spinhoven P, Ormel J, Sloekers PP, Kempen GI, Speckens AE, Van Hemert AM. (1997). A validation study of the Hospital Anxiety and Depression Scale (HADS) in different groups of Dutch subjects. Psychol Med.27(2):363-370.
64. Wylde V, Palmer S, Learmonth ID, Dieppe P. (2011). Test-retest reliability of Quantitative Sensory Testing in knee osteoarthritis and healthy participants. Osteoarthritis Cartilage.19(6):655-658.
